# Mortality rate and other clinical features observed in Open vs closed format intensive care units: A systematic review and meta-analysis

**DOI:** 10.1097/MD.0000000000016261

**Published:** 2019-07-05

**Authors:** Qian Yang, Jin Long Du, Feng Shao

**Affiliations:** Department of Intensive Care Unit (ICU), Jingzhou Central Hospital, The Second Clinical Medical College, Yangtze University, Jingzhou, Hubei, PR China.

**Keywords:** arterial line, closed format, intensive care unit, mechanical ventilation, mortality, open format

## Abstract

**Background:**

Nowadays most of the intensive care units (ICUs) operate as a closed format in comparison to an open format. The new concept of a closed ICU is where patients are admitted under the full responsibility of a trained intensivist, whereas an open ICU is where patients are admitted under the care of another attending physician and intensivists are just available for consultation. In this analysis, we aimed to systematically compare mortality rate and other clinical features observed in open vs closed ICU formats.

**Methods:**

Biomedical and pharmacological bibliographic database Excerpta Medica database (EMBASE), Medical Literature Analysis and Retrieval System Online (MEDLINE), the Cochrane Central and www.ClinicalTrials.gov were searched for required English publications. Mortality, the frequency of patients requiring mechanical ventilation, central line, arterial line and pulmonary arterial catheter were assessed respectively. Statistical analysis was carried out by the RevMan software. Odds ratios (OR) with 95% confidence intervals (CIs) were used to represent the data following analysis.

**Results:**

Five studies with a total number of 6160 participants enrolled between years 1992 to 2007 were included. Results of this analysis showed that mortality rate was significantly higher in the open format ICU (OR: 1.31, 95% CI: 1.17–1.48; *P* = .00001) (using a fixed effect model) and (OR: 1.31, 95% CI: 1.09–1.59; *P* = .005) (using a random effect model). Closed format ICUs were associated with significantly higher number of patients that required central line (OR: 0.56, 95% CI: 0.34–0.92; *P* = .02). Patients requiring mechanical ventilation (OR: 1.08, 95% CI: 0.65–1.78; *P* = .77), patients requiring arterial line (OR: 1.05, 95% CI: 0.49–2.29; *P* = .89) and patients requiring pulmonary arterial catheter (OR: 0.86, 95% CI: 0.40–1.87; *P* = .71) were similar in the open vs the closed setting.

**Conclusion:**

This analysis showed that mortality rate was significantly higher in an open as compared to a closed format ICU. However, the frequency of patients requiring mechanical ventilation, arterial line and pulmonary arterial catheter was similarly observed. Larger trials are expected to further confirm those hypotheses.

## 1 Introduction

Intensive care units (ICUs) are reserved for critically ill patients and they are vital in a hospital. Unfortunately researches on ICU patients are scarce. Nowadays different ICUs have been set up including medical,^[1]^ surgical,^[2]^ cardiac,^[3]^ neonatal,^[4]^ open and closed ICUs. Admissions in ICUs have considerately increased during the recent years. Taking into account the patients’ conditions, co-morbidities, the facilities available, the hospital or physician practice level difference and the risks of nosocomial infections, in-hospital mortality rate might apparently be predicted.^[5]^ Most of the ICUs around the world nowadays operate as a closed format with the exception of United States which still includes mainly open ICUs. In the year 2007, a survey showed that among 1265 ICUs within 75 different countries, only 17% were of ‘open’ format whereas 83% were of ‘closed’ format.^[6]^

The new concept of an open ICU is where patients are admitted under the care of another attending physician and intensivists are just available for consultation.^[7,8]^ The primary physicians or surgeons have a better familiarity with the patients and they are the main leaders involved in the management of the patients until they have completely recovered. A closed ICU, which is more common, is one where patients are admitted under the full responsibility of a trained intensivist.^[8,9]^ Advantages of a closed ICU are: focused critical care skills into a critical care environment, with better coordination, better leadership and a more cohesive treatment and a better use of resources. Even if there is evidence supporting the fact that closed format ICUs are better in terms of quality and outcomes, controversies still exist between these 2 ICU formats.

In this analysis, we aimed to systematically compare mortality rate and other clinical features observed in open vs closed ICU formats.

## 2 Methods

### 2.1 Search databases and search strategies

The PRISMA guideline^[10]^ was followed during this search process. Biomedical and pharmacological bibliographic database Excerpta Medica database (EMBASE), Medical Literature Analysis and Retrieval System Online (MEDLINE), the Cochrane Central and www.ClinicalTrials.gov were searched for corresponding English publications before September 2018 using the following searched terms:

1. ‘ intensive care unit and mortality’;
2. ‘intensive care unit and death’;
3. ‘open intensive care unit and mortality’;
4. ‘closed intensive care unit and mortality’;
5. ‘intensive care unit and clinical outcomes’;
6. ‘open ICU vs closed ICU’;
7. ‘open intensive care vs closed intensive care’;
8. -‘open ICU format vs closed ICU format’.

### 2.2 Inclusion and exclusion criteria

Studies satisfied our inclusion criteria if:

1. They were randomized trials or observational studies comparing clinical features observed in an open vs a closed format ICU;
2. They consisted of data which could be used in this analysis.

Studies satisfied our exclusion criteria since:

1. They were review articles, case studies and letters to editors;
2. They did not compare the clinical features observed in an open vs closed format ICU;
3. They consisted of data which could not be used in this analysis;
4. They were duplicated studies.

### 2.3 Endpoints which were reported

The endpoints which were reported in the original studies have been listed in Table 1.

**Table 1:**
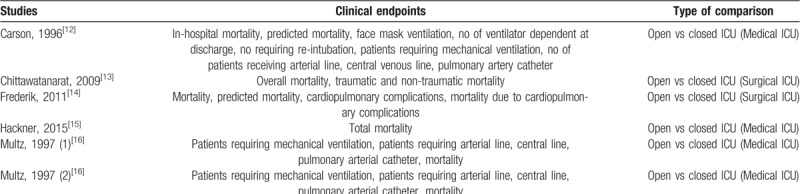
Outcomes which were assessed.

In this analysis, we were able to assess:

1. Mortality;
2. The frequency of patients requiring mechanical ventilation;
3. The frequency of patients requiring arterial line;
4. The frequency of participants requiring central line;
5. The frequency of participants requiring pulmonary arterial catheter.

### 2.4 Data extraction and review

The total number of mortality, participants requiring mechanical ventilation, participants requiring arterial and central lines, participants requiring pulmonary arterial catheter, the baseline features of the participants and data representing the main features of the studies which were included in this analysis were carefully extracted and reviewed by 3 independent reviewers.

Any disagreement which followed was further discussed and resolved by the corresponding author.

The methodological quality assessment (all the studies were observational cohorts) was carried out by the Newcastle-Ottawa Scale (NOS),^[11]^ where scores were given from 1 to 9 points, whereby a higher score represented a lower risk of bias.

### 2.5 Statistical analysis

Statistical analysis was carried out by the latest version of the RevMan software (5.3). Odds ratios (OR) with 95% confidence intervals (CIs) were used to represent the data following analysis.

Heterogeneity was assessed by the Q statistic test and the I^2^ statistic test.

A *P* value less or equal to .05 was considered statistically significant whereas for the I^2^ value, a higher percentage denoted an increased heterogeneity.

A fixed effect (I^2^ < 50%) and a random effect (I^2^ > 50%) were used based on the I^2^ value which was obtained.

Sensitivity analysis was also carried out.

Publication bias was visually observed through funnel plots.

### 2.6 Ethical approval

Ethical approval was not required for this type of study.

## 3 Results

### 3.1 Search outcomes

A total number of 45,678 publications were obtained. A careful assessment of the titles and abstracts resulted in the elimination of 45,644 articles. Thirty four (34) full text articles were assessed for eligibility.

Another assessment of the full text articles was carried out and further studies were eliminated based on the following:

1. Review articles or letters to editors (3);
2. Did not report the expected endpoints (3);
3. Did not consist of a control group (10);
4. Duplicated studies (13).

**Figure 1:**
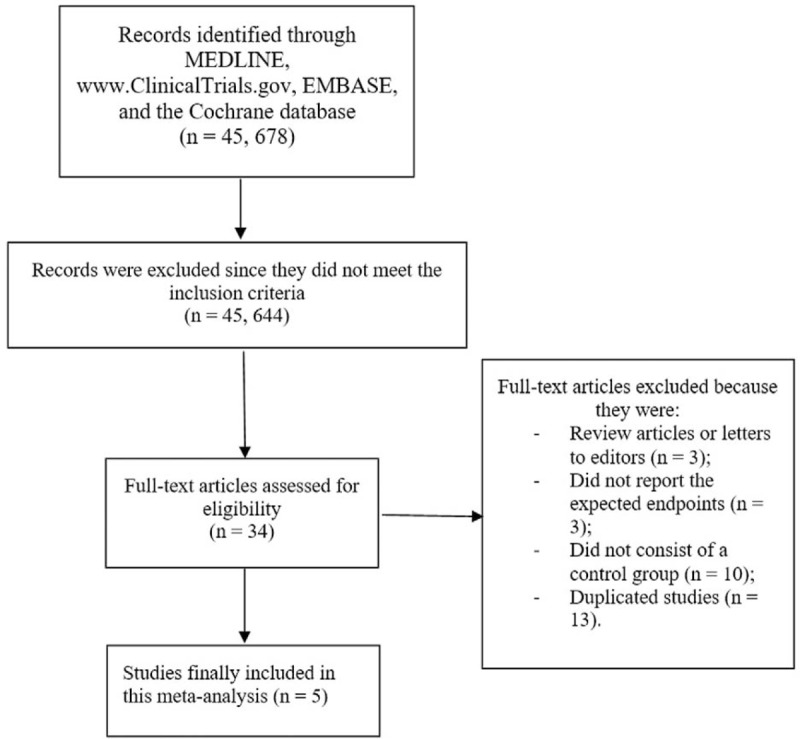
Flow diagram showing the study selection.

Finally, only 5 articles^[9,12–15]^ met the inclusion criteria and were finally selected for this analysis as shown in Figure 1.

### 3.2 General and baseline features

Five studies with a total number of 6160 participants enrolled between years 1992 to 2007 were included in this analysis (3030 participants were assigned to an open ICU format and 3130 participants were assigned to a closed ICU format) as shown in Table 2.

**Table 2:**
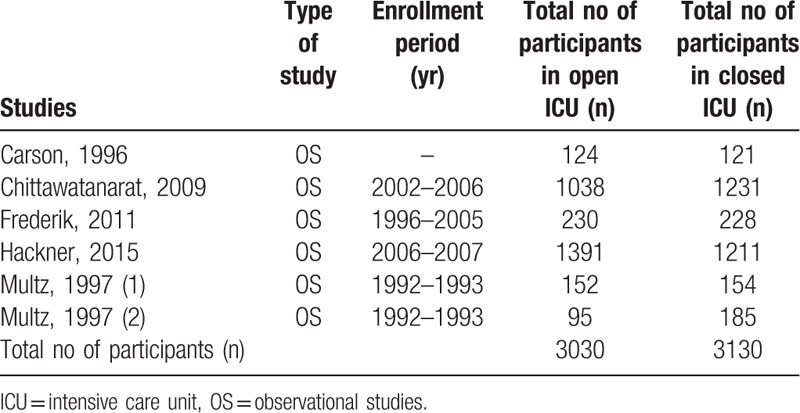
Main features of the studies.

The methodological quality assessment report has also been listed in Table 2. The total score for each study was on 9 points.

The baseline features of the participants have been listed in Table 3. The participants had a mean age ranging from 53.0 to 75.0 years. Male participants were dominant in comparison to the opposite gender.

**Table 3:**
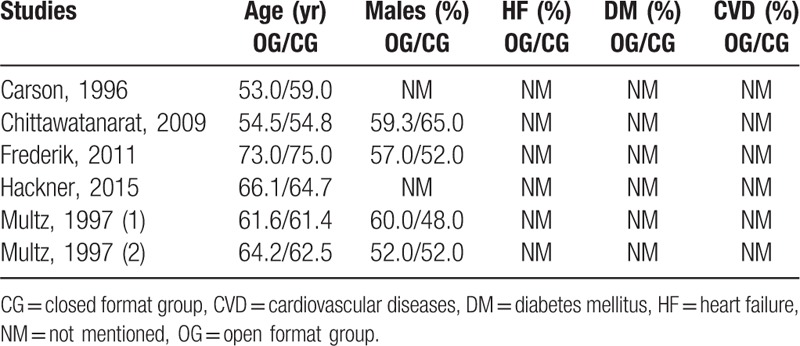
Baseline features of the participants.

### 3.3 Main results of this analysis

**Figure 2:**
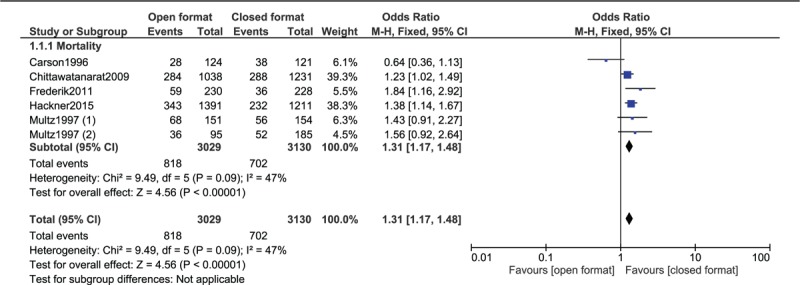
Mortality rate observed in an open vs closed ICU setting using a fixed effect statistical model.

**Figure 3:**
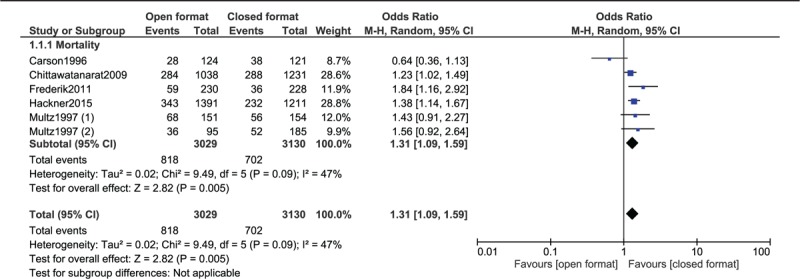
Mortality rate observed in an open vs closed ICU setting using a random effect statistical model.

Results of this analysis showed that mortality rate was significantly higher in the open format ICU (OR: 1.31, 95% CI: 1.17–1.48; *P* = .00001) [using a fixed effect model] as shown in Figure 2 and (OR: 1.31, 95% CI: 1.09–1.59; *P* = .005) [using a random effect model] as shown in Figure 3.

**Figure 4:**
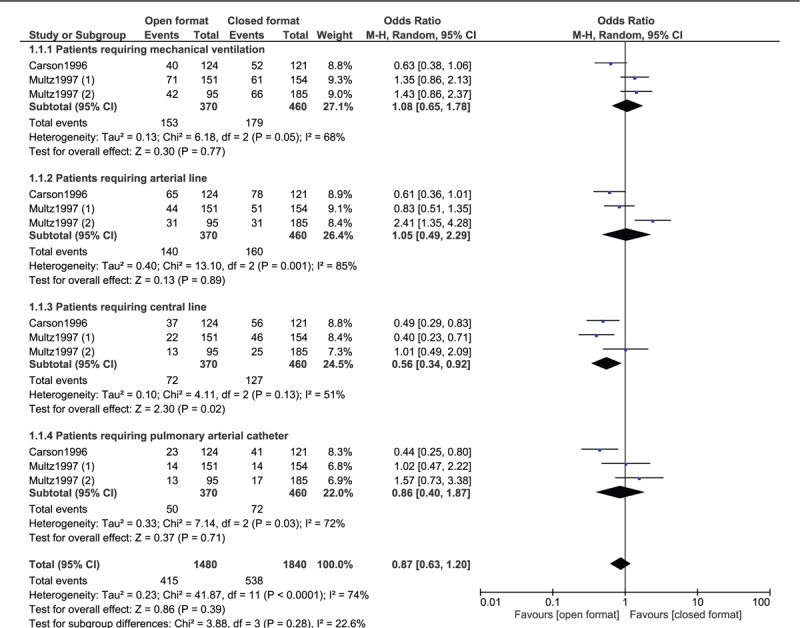
Other clinical features observed in an open vs closed ICU setting.

Patients requiring central line were significantly higher in the closed format ICU (OR: 0.56, 95% CI: 0.34–0.92; *P* = .02). Patients requiring mechanical ventilation (OR: 1.08, 95% CI: 0.65–1.78; *P* = .77), patients requiring arterial line (OR: 1.05, 95% CI: 0.49–2.29; *P* = .89) and patients requiring pulmonary arterial catheter (OR: 0.86, 95% CI: 0.40–1.87; *P* = .71) were similar in the open vs the closed setting as shown in Figure 4.

The results have been summarized in Table 4.

**Table 4:**
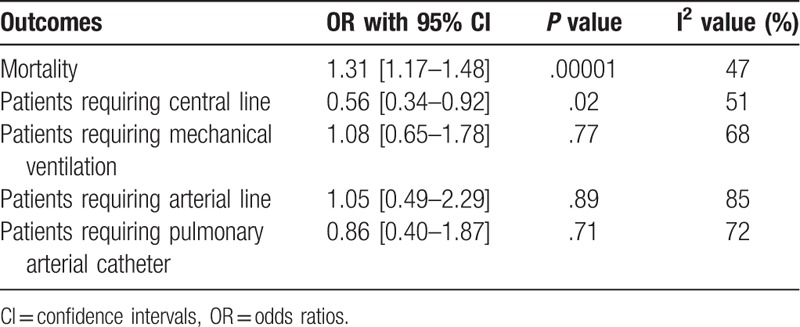
Results of this analysis.

**Figure 5:**
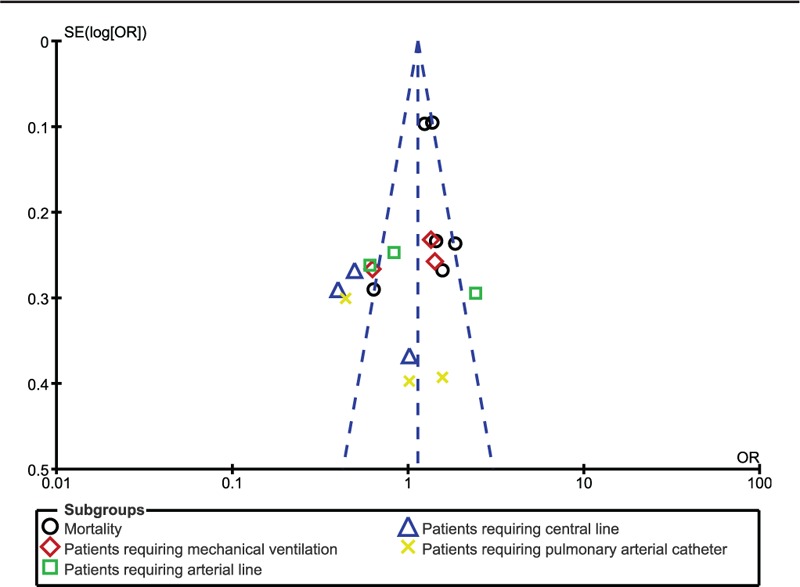
Funnel plot showing publication bias.

Sensitivity analysis was carried out and consistent results were obtained throughout. Publication bias was visually assessed using funnel plot with minor evidence of bias across the studies which assessed the endpoints (Fig. 5).

## 4 Discussion

In this analysis, mortality was significantly higher in the open format ICU compared to the closed format one. The frequency for central line was significantly higher in the closed format setting whereas the frequency for mechanical ventilation, arterial line and pulmonary arterial catheter were similarly observed in both settings.

Admission to ICU has increased recently. A recent cross-sectional, French nationwide population-based study showed variation in ICU admission among patients with cardiac disorders including heart failure.^[16]^ The authors also specified that admission for heart failure might be more prone to unwarranted variations due to medical patterns and hence, monitoring in ICU admission rate might contribute to a better insight.

Similar to this analysis, other studies showed in-hospital mortality rates for trauma,^[17]^ surgical^[13]^ and cancer patients^[18]^ admitted to a closed format ICU were significantly lower. Decreased surgical morbidity was also observed in a closed format where better care could be provided by the intensivist and team.^[19]^ Short hospital stay and well as longer duration of stay at home prior to re-admission were also observed with the closed format ICU as compared to an open one.^[20]^ However, when adjusted for severity of disease, no significant difference in cost was observed between the open and closed format ICU.^[9]^

Our results proved that intensivist-led patient management is associated with a significantly lower mortality rate and this closed model of ICU should ensure that patients and their families receive good communications, better treatment, and better expectations with better satisfaction.^[21]^

However, as suggested in a systematic review, physician staffing patterns also have an immense influence on the clinical outcomes especially on mortality of these critically ill patients too.^[22]^ High intensity staffing contributed to better outcomes and lower mortality rates.

## 5 Limitations

Due to the limited total number of participants, the results might have been affected and have been categorized by a small study effect. It should be made clear that small trials with less number of participants might have the tendency to report larger beneficial effects compared to larger trials in critical care medicine and the explanation given for this could be the poor methodological quality reported in these small trials.^[23]^ In addition, all the studies were observational cohorts which do not normally involve the best quality data. However, we could not improve this limitation due to the unavailability of randomized trials comparing open vs closed format ICUs. Another limitation was the fact that only a few studies were included during analysis of several subgroups. Also, this analysis was conducted in ICUs located in different regions, which might not be similar. Moreover, the co-morbidities among the participants were different and not clearly reported in the original studies. This might have a major impact on the outcomes reported. In addition, medical and surgical ICUs were combined and analyzed. This might also be another limitation in this study. At last, in most of the original studies, it was not mentioned whether the ICUs were covered by intensivists or in-house residents for a 24/7 period.

## 6 Conclusion

This analysis showed that mortality rate was significantly higher in an open as compared to a closed format ICU. However, the frequency of patients requiring mechanical ventilation, arterial line and pulmonary arterial catheter was similarly observed. Larger trials are expected to further confirm those hypotheses.

## Author contributions

QY, JLD and FS were responsible for the conception and design, acquisition of data, analysis and interpretation of data, drafting the initial manuscript and revising it critically for important intellectual content. QY and JLD wrote the final manuscript.

Dr Qian Yang and Dr Jin Long Du are co-first authors and they contributed equally to this paper. From the department of Intensive Care Unit (ICU), Jingzhou Central Hospital, Jingzhou, Hubei, PR China.

**Conceptualization:** Qian Yang, Jin Long Du, Feng Shao.

**Data curation:** Qian Yang, Jin Long Du, Feng Shao.

**Formal analysis:** Qian Yang, Jin Long Du, Feng Shao.

**Funding acquisition:** Qian Yang, Jin Long Du, Feng Shao.

**Investigation:** Qian Yang, Jin Long Du, Feng Shao.

**Methodology:** Qian Yang, Jin Long Du, Feng Shao.

**Project administration:** Qian Yang, Jin Long Du, Feng Shao.

**Resources:** Qian Yang, Jin Long Du, Feng Shao.

**Software:** Qian Yang, Jin Long Du, Feng Shao.

**Supervision:** Qian Yang, Jin Long Du, Feng Shao.

**Validation:** Qian Yang, Jin Long Du, Feng Shao.

**Visualization:** Qian Yang, Jin Long Du, Feng Shao.

**Writing – original draft:** Qian Yang, Jin Long Du.

**Writing – review & editing:** Qian Yang, Jin Long Du.
